# Single-cell RNA sequencing analysis of vestibular schwannoma reveals functionally distinct macrophage subsets

**DOI:** 10.1038/s41416-024-02646-2

**Published:** 2024-03-13

**Authors:** Paramita Baruah, Christopher Mahony, Jennifer L. Marshall, Charlotte G. Smith, Peter Monksfield, Richard I. Irving, Ingrid E. Dumitriu, Christopher D. Buckley, Adam P. Croft

**Affiliations:** 1 Department of ENT, University Hospitals of Birmingham NHS Trust, Birmingham, UK; 2 Department of ENT, University Hospitals of Leicester NHS Trust, Leicester, UK; 3 Institute of Inflammation and Ageing, University of Birmingham, Birmingham, UK; 4 Institute of Cardiovascular Sciences, University of Birmingham, Birmingham, UK

**Keywords:** Tumour immunology, Oncology

## Abstract

**Background:**

Vestibular schwannomas (VSs) remain a challenge due to their anatomical location and propensity to growth. Macrophages are present in VS but their roles in VS pathogenesis remains unknown.

**Objectives:**

The objective was to assess phenotypic and functional profile of macrophages in VS with single-cell RNA sequencing (scRNAseq).

**Methods:**

scRNAseq was carried out in three VS samples to examine characteristics of macrophages in the tumour. RT-qPCR was carried out on 10 VS samples for CD14, CD68 and CD163 and a panel of macrophage-associated molecules.

**Results:**

scRNAseq revealed macrophages to be a major constituent of VS microenvironment with three distinct subclusters based on gene expression. The subclusters were also defined by expression of CD163, CD68 and IL-1β. AREG and PLAUR were expressed in the CD68+CD163+IL-1β+ subcluster, PLCG2 and NCKAP5 were expressed in CD68+CD163+IL-1β− subcluster and AUTS2 and SPP1 were expressed in the CD68+CD163−IL-1β+ subcluster. RT-qPCR showed expression of several macrophage markers in VS of which CD14, ALOX15, Interleukin-1β, INHBA and Colony Stimulating Factor-1R were found to have a high correlation with tumour volume.

**Conclusions:**

Macrophages form an important component of VS stroma. scRNAseq reveals three distinct subsets of macrophages in the VS tissue which may have differing roles in the pathogenesis of VS.

## Introduction

Vestibular schwannomas (VSs) are benign primary intracranial tumours of the myelin-forming cells of the eighth cranial nerve but can cause considerable morbidity (stroke, cranial nerve palsies with speech and swallowing impairment, facial palsy, hearing loss), sometimes with fatal outcomes. Surgery and/or radiotherapy for the treatment of growing VS is associated with significant morbidity and mortality and is therefore reserved for tumours demonstrating growth on imaging, large (>2–3 cm) or at a size sufficient to cause intracranial pressure symptoms. Growth of VS and its size demonstrated by imaging is the determining criterion in its management and prognosis. It is not clear, however, what cellular and molecular mechanisms drive the tumour growth in VS. Understanding these mechanisms will allow development of new treatment modalities to arrest or reverse tumour growth.

Tumour stroma, and in particular tumour-associated macrophages (TAMs), play a crucial role in regulating tumour progression by supporting angiogenesis, tumour cell proliferation, invasion, metastasis and mechanisms of resistance to treatment [1]. Presence of macrophages in VS tissue has been shown previously and a link between macrophage infiltration and the size of the tumour and hearing outcomes has been suggested [2, 3]. Macrophages are important innate immune cells that are associated with two distinct phenotypes: a proinflammatory (or classically activated) subset with functions such as inflammatory cytokine production and bactericidal activity and an anti-inflammatory (or alternatively activated) subset linked with wound healing and tissue repair [1]. Several factors influence the recruitment, differentiation and function of macrophages in the tumour microenvironment, and this is a subject of active interrogation in several cancers.

The functional profile of the macrophages in VS tissue has not been explored in detail. This is an important facet to examine as VS growth could be influenced not just by the number of infiltrating macrophages but also the functional status of the TAMs. In this study, we confirm the presence of macrophages in situ in VS tissue using immunofluorescence. We show using RT-qPCR that VS tissue exhibits expression of several molecules which can impact on macrophage function. We further correlate the expression of these macrophage related molecules with the size of VS. Of note, we perform single-cell RNA sequencing (scRNAseq) on VS tissue and show novel data identifying three subsets of macrophages in VS with distinct functional profiles. Further understanding of macrophage subsets and their functional status in VS will unveil newer dimensions of disease pathogenesis and potentially reveal new therapeutic targets to improve patient outcomes.

## Materials and methods

### Patient demographics

Ethical approval for the study was obtained from the institute’s research ethics committee (Human Biomaterials Resource Centre HBRC 17-295) and the tissue samples were released via HBRC. All methods were performed in accordance with the relevant guidelines and regulations.

Patients were recruited into the study following informed consent. VS tissue collected from 13 patients undergoing excision of the tumour has been included in this study. Tumour size was obtained from the pre-operative imaging (CT scan/MRI scan). Patients’ details are summarised in Table 1.

**Table 1:** Patient demographics.

Patient number	Age	Gender	Vol. of tumour (mm^3^)	Surgery	Type
AN001	44	F	61,440	Translab	Cystic
AN002	46	M	14,812	Retrosig	Solid
AN003	72	F	39,701	Translab	Solid
AN004	51	F	43,000	Translab	Solid
AN005	53	F	51,200	Translab	Solid
AN006	76	F	28,672	Translab	Solid
AN007	46	F	39,060	Translab	Solid
AN010	65	M	10,560	Retrosig	Cystic
AN011	52	F	13,392	Translab	Cystic
AN012	68	F	65,120	Translab	Cystic
AN014	31	M	20,677	Translab	Solid
AN017	33	F	32,591	Translab	Solid
AN018	35	M	45,954	Translab	Cystic

*Translab* translabyrinthine approach, *Retrosig* retrosigmoid approach.

### Human tissue processing and histology

Details are presented in Supplementary Methods.

### Quantitative reverse transcription PCR

RNA was isolated from frozen tissue using the RNAeasy RNA isolation kit (Qiagen) according to the manufacturer’s instructions. cDNA synthesis was performed on all samples (500 ng of RNA was transcribed) using SensiFAST cDNA Synthesis Kit (Bioline) on a Mastercycler (Eppendorf) thermal cycler PCR machine. Reverse transcription with quantitative PCR (RT−qPCR) was performed using a Taqman Gene Expression array and Taqman universal Mastermix on the ABI 7900 real-time PCR detection system (both Applied Biosystems) using the TaqMan Array Microfluidic Card. Expression levels were normalised to an internal housekeeping gene (GAPDH) and a relative amount of expression for genes of interest was calculated form the delta CT to the housekeeping gene (2^−ΔCT^). The primers used in the arrays were for the following genes of interest: STAT-1, ALOX15, INHBA, CCL2, CCL5, IL8, CxCL10, CD64, SPl1, CD32, IL6, IL10, TNF, IL1b, RANK, MRC1, PTPRC, EpCAM, ACP5, CTSK, CD68, RPL13A, MERTK, HLADRA, CD163, CD14, FN1, Thy1, PDPN, CD80, CD16b, MCT4, MCT3, Cav1, HIF1a, CSF1R, CSF2RA and IDO.

### Single-cell RNA analysis

#### Enzymatic digestion of human tumour tissue

Tumour tissue samples were disaggregated into single-cell suspension as previously described for synovial tissue [4]. In brief small fragments (~1–2 mm^3^) of tumour tissue were generated by dissecting with forceps or surgical scissors. The cut fragments were cryopreserved for subsequent disaggregation; they were transferred to a cryovial (1.5 ml; Nalgene) containing 1 ml of CryoStor® CS10 for viable freezing. Tumour fragments were thawed, washed and dissociated by enzymatic digestion using RPMI media with Liberase™ TL enzyme preparations (100 μg/ml; Roche) and DNase I (100 μg/ml; Roche) prior to single-cell analysis. Single-cell suspensions were assessed for cell quantity and cell viability. To remove non-viable cells before library construction, 7-AAD viability dye (#A1310, ThermoFisher) was used.

#### Generation and analysis of droplet-based scRNAseq data

All viable cells (*n* = 3 samples, each consisted of cells isolated from VS tissue from three different patients) were captured with the 10× Genomics Chromium system. Sequencing libraries were generated using the 10x Genomics Single Cell 3′ Solution (version 3.1) kit and subjected to Illumina sequencing (NextSeq 2000). Alignment to GRCh38 was performed using the 10× Genomics Cell Ranger pipeline (v.7.0.0). Analysis was completed using and R (v4.1) and Seurat (v4.0.03) [5]. The following QC metrics were used for all samples: nFeature_RNA>200 & nFeature_RNA<6000 & mitochondrial % <10. The following Seurat functions were used to process the data: NormalizeData(), FindVaribleFeatures(), ScaleData(), RunPCA(), RunUMAP(), FindNeighbours(), FindClusters(). Samples were integrated using FindIntegrationAnchors() and IntegrateData(). Macrophages were harmonised using Harmony Package (v0.1) and HarmonyMatrix() [6]. Differential expression between clusters was calculated using FindAllMArkers() and FindMarkers() in Seurat. GO term analysis was completed using gsFisher (v0.2) package.

Data were either prepared using Microsoft PowerPoint (version 16.51) or Adobe Photoshop (v24.6.0).

### Statistical analysis

Statistical analysis was performed using GraphPad Prism software version 8.4. RT-qPCR data on VS tissue and tumour size were compared using Spearman correlation analysis. Probability values (*p*) of <0.05 were considered statistically significant.

## Results

### Patients and tissue samples

The diagnosis of VS in the patients was made on clinical grounds. Informed consent was obtained from all patients. VS samples from 13 patients were included into the study of which 3 were used for the scRNAseq and 10 were used for the RT-qPCR analysis. The age range of the patients at surgery was 31–76 years with a mean of 46.6 years; 4 of the tissue samples originated from male and 9 from female patients. Clinical data obtained included patient age at operation, gender and volume of tumour and is summarised in Table 1. The diagnosis of VS was confirmed on histopathology according to WHO criteria in the Department of Pathology, University of Birmingham Hospitals, UK. Tumours were graded according to the WHO classification of tumours of the central nervous system [7]. All the tumours in this study were classified as WHO I grade. Size of the tumour was calculated as the volume from measurements of three dimensions on the pre-operative MRI by two clinicians.

### Correlation of macrophage marker expression to VS size

**Fig. 1:**
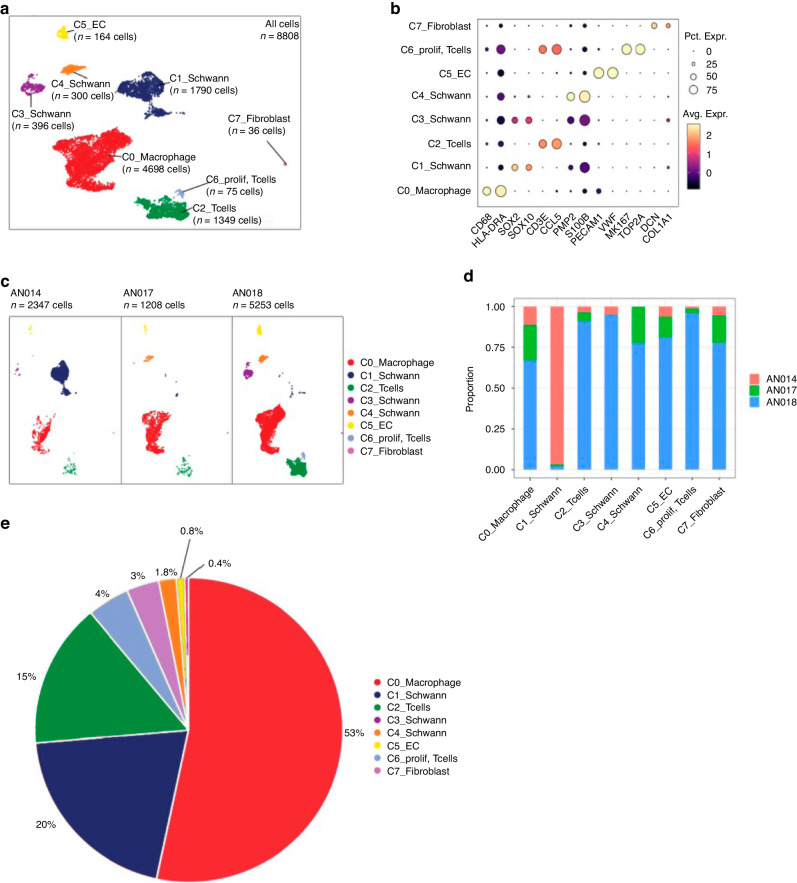
Single-cell RNA analysis of cellular subsets in vestibular schwannoma (VS). **a** The merged Uniform Manifold Approximation and Projection (UMAP) plot of 8808 VS cells from three patients (AN014, AN017 and AN018) showing the presence of 7 subclusters of cells based on expression of specific markers. **b** Expression of marker genes in the seven subclusters on scRNA analysis CD68, SOX2, CD3, PMP2, S100B, PECAM1, MK167, Decorin (DCN). **c** UMAP single-cell RNA analysis of the three patients presented individually. **d** Stacked bar chart of seven major cell-type composition of each patient. **e** Pie chart showing the composition of the VS tumour on single-cell analysis.

We first examined expression of CD163 (a marker of M2 macrophages) in tissue sections of VS tissue using immunofluorescence. Macrophage infiltration was noted in the VS tissue in keeping with previously reported findings [2] (Supplementary Fig. 1A). We then performed RT-qPCR on fresh frozen VSs from 10 patients to study a panel of macrophage-associated molecules (details in ‘Materials and methods’; Supplementary Fig. 1B). CD163, the marker of M2 macrophages was expressed in VS tissue (Supplementary Fig. 1B) on RT-qPCR in keeping with the findings on immunofluorescence (Fig. 1a). Correlation analysis of the molecules found to be expressed on RT-qPCR (Supplementary Fig. 1B) was performed with the volume of the VS (Table 1) using Spearman correlation coefficient. A strong correlation was found between the macrophage/monocyte marker CD14 (*ρ* 0.71, *p* = 0.027) and tumour volume (Table 2A). A moderate correlation (correlation factor, *ρ* 0.4) was noted between VS volume and CD163 expression (M2 marker) (Table 2B). Similarly, a moderate correlation was found between VS volume and the pan macrophage marker CD68 expression (*ρ* 0.55) (Table 2B). Strong correlation to tumour volume was also observed with ALOX15 (*ρ* 0.712, *p* = 0.02), Interleukin-1B (*ρ* 0.6, *p* = 0.07) a cytokine produced by macrophages [8]; Inhibin A (INHBA, *ρ* 0.63, *p* = 0.07) a member of the TGF-b family which is involved in myeloid cell function [9]; and Colony Stimulating Factor 1R (*ρ* 0.61, *p* = 0.06) which controls the production, differentiation and function of macrophages [10] (Table 2A). Interleukin-6, which influences M2 polarisation of macrophages [11], had a correlation coefficient of *ρ* 0.51 with tumour volume (Table 2B). No-to-low correlation was observed between VS volume and RANK (*ρ* −0.04), which is associated with osteoclasts/macrophage differentiation [12], or Indoleamine 2,3 Dioxygenase (IDO, *ρ* −0.28), which associates with M2 macrophage [13] differentiation (Table 2C). The other macrophage-associated molecules and epithelial markers examined and their respective correlation coefficients are as follows (also listed in Table 2A–C): STAT1 (0.37), IL10 (0.38), TNF (0.38), INHBA (0.6), IL8 (0.48), CCL2 (0.45), CCL5 (0.54), CD64 (0.25), MERTK (0.37), EPCAM (0.33), PTPRC (0.32), MCT3 (0.35), CSF2RA (0.34), Thy1 (0.14), RPL13A (0.11), RANK (−0.04), CD80 (−0.02), PDPN (−0.2), SPl1 (0.52), IL-8 (0.52), MRC1 (0.5), CD32 (0.44), CTSK (0.44), ACP5 (0.4), HLA-DRA (0.43), CD16b (0.43), FN-1 (0.42), MCT4 (0.53), Cav1 (0.57), HIF1A (0.53) and CxCL10 (−0.43).

**Table 2:** (A) Markers with strong correlation with tumour size; (B) markers with moderate correlation with tumour size; (C) markers with weak correlation with tumour size.

(A)	Rho	*p*
ALOX15	0.7212	0.0234
CD14	0.7091	0.0268
INHBA	0.6333	0.076
CSF1R	0.6121	0.0667
IL1B	0.6	0.0734

(B)	Rho	*p*
CD68	0.55	0.1049
CCL5	0.54	0.1139
MCT4	0.53	0.1231
IL6	0.52	0.1334
SPI1	0.52	0.1334
IL8	0.52	0.1334
MRC1	0.5	0.144
HIF1A	0.49	0.1548
CCL2	0.45	0.1912
CD32	0.44	0.2044
CTSK	0.44	0.2044
HLA-DRA	0.43	0.2182
CD16b	0.43	0.2182
FN1	0.42	0.2325
CD163	0.41	0.2475
Cav1	0.41	0.2475
ACP5	0.4	0.2912

(C)	Rho	*p*
STAT1	0.37	0.2957
IL10	0.38	0.2788
TNF	0.38	0.2788
CD64	0.25	0.4918
MERTK	0.37	0.2957
EPCAM	0.33	0.4279
PTPRC	0.32	0.3679
MCT3	0.35	0.3586
CSF2RA	0.34	0.3304
THY1	0.14	0.7072
RPL13A	0.11	0.8397
RANK	−0.04	0.9184
CD80	−0.02	0.9768
PDPN	−0.2	0.5837
IDO	−0.28	0.5008
CXCL10	−0.43	0.2992

### Identification of macrophages in VS on single-cell RNA analysis

As macrophage function-associated molecules expressed in VS were found to co-relate with tumour volume, we next performed scRNAseq of VS samples from three patients to analyse the macrophage profile of VS microenvironment at the single-cell level. Seven cellular clusters were identified on scRNAseq in VS tissue in the integrated UMAP (Fig. 1a). Cellular clusters in individual patients (Fig. 1b) AN014, AN017, AN018 showed a similar distribution as seen in the integrated UMAP with some heterogeneity noted in the proportion of cells from each donor (Fig. 1c, origin identity). Cellular clusters in the integrated UMAP were examined for expression of known marker genes and denominated accordingly (Fig. 1a, b). Cluster 0 was found to express macrophage marker CD68 [14] and therefore nominated as the macrophage cluster. Cluster 2 was denominated as T cells (expression of CD3 [15]), and cluster 6 were proliferating T cells (expression of CD3 and MK167− proliferation marker [16]). Cluster 1, 3 and 4 were all Schwann cells and possible pre-cursors. Cluster 1 was Schwann cells with strong expression of S100B [17], SOX2 [18] and PMP2 [19] (SC1). Cluster 3 was composed of Schwann cells with strong expression of S100B and lower positivity with SOX2 and PMP2 (SC2). Cluster 4 was composed of Schwann cells with strong expression of S100B and PMP2 and low levels of SOX2 (SC3). Cluster 5 was composed of endothelial cells (EC) with expression of PECAM [20] and Von Willebrand factor [21]. Cluster 7 was designated as the fibroblast cluster (expression of Collagen markers and decorin [22]). Proportion of the cells in the clusters in the integrated UMAP is shown in a pie chart (Fig. 1e) showing that immune cells (macrophages and T cells) form a significant proportion of the cells in VS. SC1 cells were noted to be predominantly from one patient (AN14) who had very few SC2 cells indicating heterogeneity among the tumours (Fig. 1c).

**Fig. 2:**
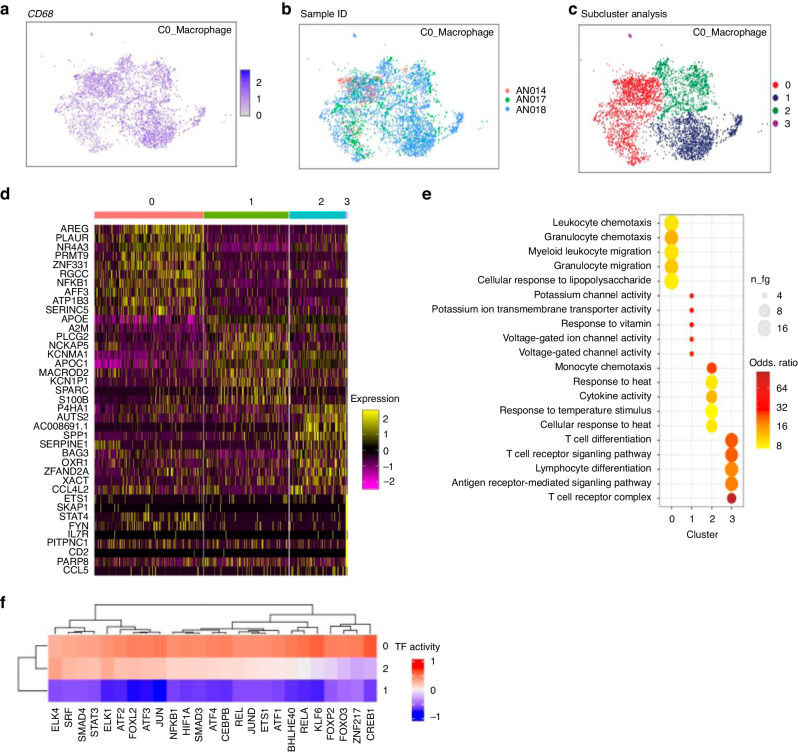
Single-cell RNA analysis of macrophage subsets in vestibular schwannoma (VS). The single-cell RNA analysis of the macrophage subset in three VS tissue samples from different patients. **a** Expression of CD68 in the macrophage subset on single-cell RNA analysis. **b** Integrated dataset showing the subclusters of macrophages based on contribution from each donor. **c** The integrated dataset showing four subclusters (red, dark blue, green and purple) of macrophages based on gene expression. **d** The analysis in the four subsets showing the ten highly expressed genes as a heat map. Cluster 0, Cluster 1 and Cluster 2 represent macrophage subsets. Cluster 3 are contaminating T cells. **e** Pathway analysis of the macrophage subsets on scRNA sequencing is shown (Go Terms). **f** DoroThea plot of the macrophage subclusters 0, 1 and 2 showing the expression of transcriptional factors in the three subsets.

We further analysed the macrophage cluster that formed 53% (Fig. 1e) of all the cells analysed and was identified by expression of CD68 (Fig. 2a). The macrophages were derived from all three donors (Fig. 2b). The identity origin of the macrophage cluster before and after batch correction is depicted in Supplementary Fig. 2A. The macrophage cluster revealed further four subclusters (Fig. 2c) based on the 10 highest expressing genes analysed in these subclusters (presented as a heatmap (Subcluster 0–3, Fig. 2d). Subcluster 3 showed high expression of T cell markers (IL7R and CD2) indicating T cell spillover/contamination and was therefore not analysed further. Subcluster 0 macrophages showed high expression of Amphiregulin (AREG), PLAUR (Urokinase plasminogen activator surface receptor) and AFF3 (AF4/FMR2 family member 3). Subcluster 1 macrophages showed high expression of PLCG2, NCKAP5 and S100B. Subcluster 2 macrophages had high expression of AUTS2, SPP1 and SERPINE1.

### Pathway analysis of the macrophage subgroups (GO Terms)

To understand the functions of each macrophage subcluster better, GO Terms analysis was performed to determine the genes that were significantly enriched in each cluster. The three subclusters fell into three distinct functional pathways (Fig. 2e).

Subcluster 0 expressed genes involved in leucocyte chemotaxis/migration and response to LPS which are well known functions of macrophages. Subcluster 1 macrophages showed expression of genes involved in potassium and voltage channel activity while Subcluster 2 expressed genes involved in monocyte chemotaxis, cytokine activity and heat response. A further analysis of highest expressed genes in the pathway analysis per subcluster is shown in Supplementary Table 1. The fourth subcluster previously designated as T cells showed genes associated with T cell function such as T cell differentiation and receptor signalling as expected. Our results indicate a functionally active profile of macrophages in Subcluster 0 and Subcluster 2. As the macrophage subclusters were functionally heterogenous, DoroThea (transcription factor) analysis of the macrophage clusters was performed next (Fig. 2f). This showed that Cluster 0 is more transcriptionally active while Cluster 1 is least transcriptionally active; which is in keeping with the GO Term analysis. Cluster 0 expressed several tumour-related transcription factors such as ELK4, CREB1, cJun, JunD, KLF6, ETS-1 and ZNF217. In addition, several transcription factors regulating inflammation were also highly expressed in Cluster 0 such as STAT3 (which inhibits anti-tumour immunostimulatory genes and upregulates genes crucial for oncogenesis and cancer inflammation) [23], Smad4 that is linked to TGF-β function [24], ATF-2 that regulates macrophage response to LPS [25], NFκb-1 (induces expression of proinflammatory genes) [26], CEBPB (regulates release of proinflammatory cytokines from THP cells) [27], BHLHE40 that promotes macrophage proinflammatory gene expression [28] and FOXO3 that influences macrophage function by negative regulation of IL-10 [29]. These results suggest that macrophages play an important role in tumourigenesis and inflammatory milieu in VS.

### A phenotypic and functional analysis of macrophage subsets in VS based on CD68, CD163 and IL-1β expression

**Fig. 3:**
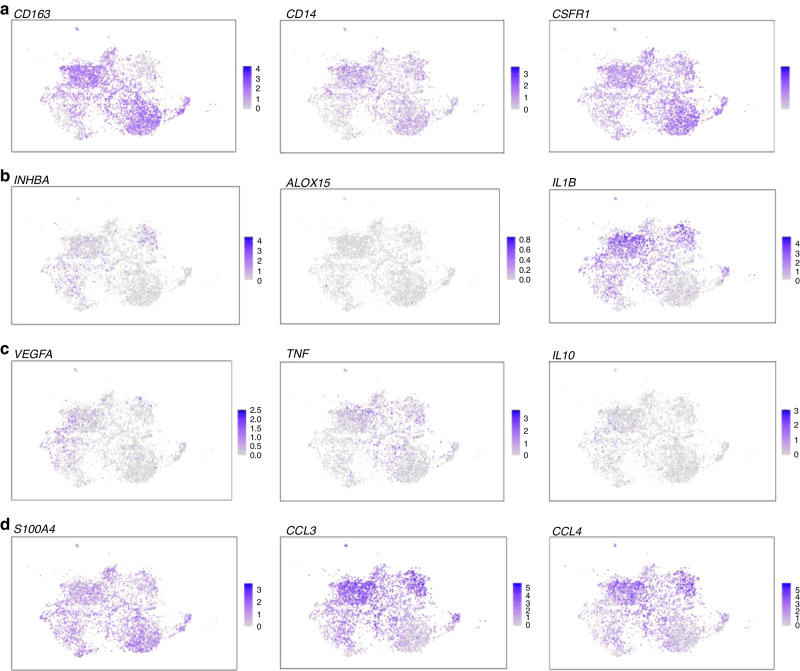
Expression of CD163 and IL-1β in macrophage subsets in vestibular schwannoma (VS) on scRNA analysis. Single-cell RNA analysis of the macrophage subsets in three vestibular schwannoma tissue samples from different patients. **a** Expression of CD163, CD14 and CSFR1 in the macrophage subsets. **b** Expression of INHB-A, ALOX-15 and IL-1β in the macrophage subsets. **c** Expression of VEGF-A, TNF-α and IL-10 in the macrophage subsets. **d** Expression of TMEM119, CCL3 and CCL4 in the macrophage subsets.

We next evaluated the subclusters for the expression of M2 macrophage marker CD163 **(**Fig. 3). Of note while CD163 was expressed in subclusters 0 and 1, it was absent in cluster 2. We next examined the expression of the markers identified previously (Table 2A) that showed good correlation with tumour volume -ALOX15, IL-1β, CSFR1, CD14 and INHBA - to their expression at the single-cell level in the macrophage subclusters. CSFR1 and CD14 expression were present in all three macrophage subclusters (Fig. 3a). INHBA expressing cells were noted in subclusters 0 and 2 while ALOX15 expression could not be ascertained at a single-cell level, potentially due to limitations of the scRNA analysis (Fig. 3b). Interestingly, IL-1β was found to be well expressed in subclusters 0 and 2 (Fig. 3b). Of note, we found that the three subclusters of VS macrophages identified via the scRNA analysis could also be classified based on the expression of CD68, CD163 and IL-1β (Subcluster 0 = CD68+CD163+IL-1β+, Subcluster 1 = CD68+CD163+IL-1β− and Subcluster 2 = CD68+CD163−IL-1β+).

The three macrophage subsets identified above based on CD68, CD163 and IL-1β expression were further interrogated for a range of phenotypic and functional macrophage markers on the scRNA analysis—these included Tumour necrosis factor-alpha (TNF-α), Interleukin 10 (IL-10), Interferon-gamma (IFN-γ), Vascular endothelial growth factor A (VEGF-A), MER proto-oncogene tyrosine kinase (MERTK), S100 calcium binding protein A4 (S100A4), Glial Fibrillary acidic protein (GFAP), Marker of Proliferation Ki-67 (MK167), Tyro3 (protein tyrosine kinase, Axl (tyrosine kinase), CCL2(MCP-1), CCL3, CCL4, CCL5, P2RY12, Siglec15, and signal regulatory protein alpha (SIRPα). Further differences were noted in the cytokine profile of the macrophage subclusters. CD68+CD163+IL-1β+ macrophages expressed TNF-a and IL-10. CD68+CD163−IL-1β+ macrophages and CD68+CD163-IL-1β− macrophages expressed TNF-a but not IL-10 (Fig. 3c, d). Of note, VEGF-A expression was primarily noted in the CD68+CD163+IL-1β+ macrophages (Fig. 3c). S100A4, CCL3, CCL4, and MERTK expression was found in all three subsets of macrophages (Fig. 3d and Supplementary Table 2). Expression of the above and additional macrophage related markers in the three subclusters is summarised in Supplementary Table 2. The expression of these markers in the whole UMAP for comparison to the expression in the macrophage cluster is shown in Supplementary Fig. 3A. In addition, we analysed Iba1 (ionised calcium binding adaptor molecule 1, macrophage/microglial marker) which was found to be expressed in the macrophage subcluster while expression of CD206 and CD80 was low (Supplementary Fig. 3B–D).

**Fig. 4:**
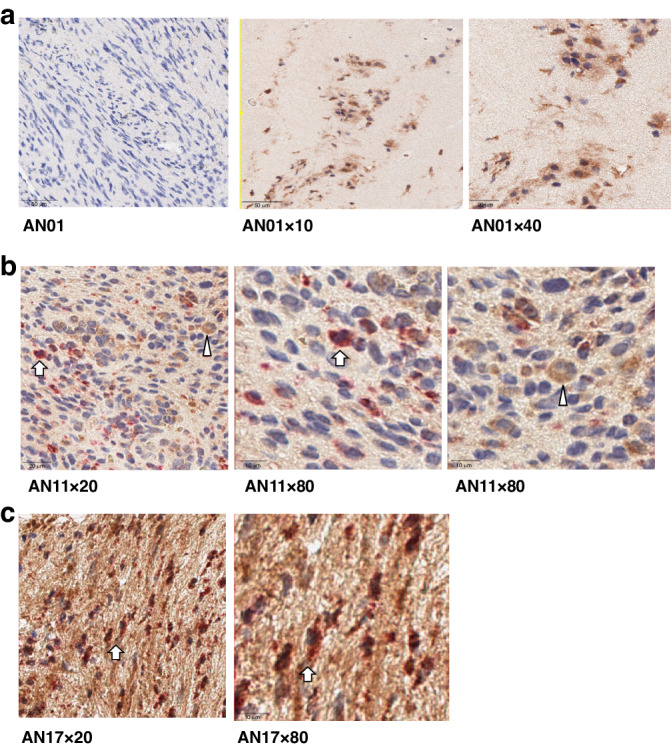
Expression of CD68, CD163 and IL-1β in vestibular schwannoma (VS) tissue on immunohistochemistry. Immunohistochemistry images of VS tissue (representative of *n* = 8 VS patient samples). **a** IL-1β is in brown, nuclei are blue (middle and right panels), left-hand side tissue section depicts control staining. **b** CD163 is in pink and IL-1β is in brown. Middle and right panels are enlarged images taken from the areas indicated by the arrow/arrowhead on the left-hand side tissue section showing CD163/IL-1β double co-expressing macrophage (arrow) and IL-1β expressing but CD163 negative cell (arrowhead), respectively. **c** CD68 is in pink and IL-1β is in brown. Right side panel is image is taken from the area indicated by the black box on the left-hand side tissue section showing CD68 and IL-1β expressing macrophages.

We next examined the expression of IL-1β in VS tissue on immunohistochemistry. A strong cellular expression of IL-1β was noted in all VS tissue examined (Fig. 4a). Further co-staining with CD163 or CD68 along with IL-1β confirmed that macrophages in VS tissue express IL-1β (Fig. 4b, c). A higher background was observed with the double staining experiments than with IL-1β alone and is likely to be non-specific.

### Comparison of VS macrophages with macrophages in glioblastoma multiforme (GM)

We next compared the macrophage clusters in VS to the macrophage clusters described in GM by Cui et al. [30]. The macrophages in GM were identified via the expression of CD68 as we did in VS. TMEM119 was used to distinguish between microglia (brain tissue-derived macrophages), which express TMEM119, versus bone marrow-derived macrophages which do not. In GM, the percentage of tumour associated macrophages was 36.39%, with microglia versus macrophages components being 16.10% versus 20.29% respectively [30]. In VS, the macrophage cluster comprised 53% of the entire cellular population (Fig. 1e). Within the VS macrophage cluster, the expression of TMEM119 was low and noted mainly in Cluster 2 (CD68+CD163−IL-1β+ macrophage subcluster) (Supplementary Fig. 2). The gene profile analysis of macrophages in GM indicated the presence of priming macrophages: defined by the expression of cell cycle-associated genes [aurora kinase B (AURKB), cell division cycle associated 3 (CDCA3), and assembly factor for spindle microtubules (ASPM). HLA-positive macrophages were classified as primed macrophages [30]. HLA-negative macrophages were categorised as repressed, due to the expression of Metallothionein (MT1G) and ankyrin repeat domain 28 (ANKRD28). We therefore examined the expression of the above genes in the VS macrophage clusters. The cell-cycle associated genes AURKB, CDCA3 and ASPM were all very low in the VS macrophage subclusters. HLA-DQA2 was expressed in all three VS macrophage cluster with the maximum expression in Cluster 1 and lowest in Cluster 2. HLA-DQB2 was expressed at low levels in all three macrophage subclusters as was MT1G. In contrast, the highest expression of ANKRD28 was in Cluster 0 macrophages with negligible expression in the other macrophage clusters.

## Discussion

This study presents the functional profile of macrophages in stromal microenvironment in VS and in-depth analysis of macrophages in the VS tissue using scRNAseq. We show that expression of classical macrophage-related molecules such as CD14, CD163, CD68 and several molecules involved in the function, differentiation and recruitment of macrophages in VS tissue using RT-qPCR, many of which show a correlation to VS tumour volume. We also present novel findings from the scRNA sequencing of three distinct macrophage subpopulations in the VS microenvironment with distinct functional and activation profiles.

Macrophages act as immunoregulators in tumour progression and within the tumour microenvironment undergo phenotypic polarisation. M1 or classically activated macrophages have phagocytic and anti-tumour inflammatory reactions. In contrast, M2 (alternatively) activated macrophages have tumour-promoting abilities, such as immunosuppression and angiogenesis [31]. While macrophage presence and their roles in pathogenesis of malignant tumours has been extensively studied, this is not the case with pathologically benign tumours such as VS. We and others confirm the presence of CD163 expressing macrophages in VS tissue [2]. Previous reports also indicate that infiltration with CD163 expressing macrophages correlates with tumour size in VS [2]. We show that macrophage markers CD68, CD163 and CD14 are expressed in VS on RT-qPCR. Of these, CD14 correlated strongly with the volume of VS while CD163 (a marker for M2 differentiation of macrophages) and CD68 (a pan macrophage marker), both showed moderate correlation with VS volume. Our results are in keeping with previous studies indicating that macrophage infiltration links to tumour size [2, 32] Some studies suggest that macrophage infiltration can also associate with tumour growth [21] while others indicate that while macrophages and lymphocytes are linked to tumour volume they do not necessarily link to tumour growth [33] (also summarised in Hannan et al. [34]).

The above discrepancy in reported literature suggests that the functional profile of infiltrating macrophages in VS and factors driving recruitment of macrophages into VS may also be the drivers of tumour growth rather than the number of infiltrating macrophages alone. However, the functional profile of macrophages in VS is yet to be completely elucidated. Our RT-qPCR data in VS tissue shows the expression of several macrophage markers in VS tissue, including cytokines that influence macrophage function such as IL-1β and IL-6. IL-1β in particular, showed a strong correlation to VS volume. IL-1β is produced by macrophages and in tumour microenvironment dominated by tumour associated macrophages has been shown to promote tumour growth and metastasis in breast cancer [35] and may have implications in VS growth as well. Of note, a meta-analysis of VS microarray data showed a correlation between NLRP3 inflammasome and hearing loss in VS irrespective of tumour size [36]. This paper also observed that increased IL-1β expression in VS tissue trended towards poorer hearing. This is of interest as NLRP3 inflammasome activates pro-IL-1β to activate IL-1β (reviewed in Blevins et al. [37]) and therefore both may represent interesting therapeutic targets. IL-6 expression showed moderate correlation with tumour size. IL-6 is a pleiotropic cytokine and can promote M2 polarisation of macrophages in sites of inflammation and tumour microenvironment [11, 38] and could have a role in the M2 skewed profile of macrophage infiltration in VS. An interesting finding in our work was the expression of ALOX15 and its strong correlation to VN tumour size. Lipoxygenases (LOXs) are non-haem iron-containing dioxygenases that catalyse the stereo-specific peroxidation of polyunsaturated fatty acids to hydroperoxy derivatives. Resolution phase macrophages are highlighted by the strong up-regulation of arachidonate 15- lipoxygenase (ALOX15), a key enzyme involved in the synthesis of specialised pro-resolving mediators including lipoxins (LXs), resolvins (Rvs), protectins, and maresins that facilitate inflammation resolution [39]. Future work may reveal interesting facets about ALOX15 in VS pathogenesis. The scRNAseq did not elicit ALOX15 expression which may be within the technical limitations of the sequencing process.

Inhibin beta A (INHBA) also showed a strong correlation to VS size. It belongs to the transforming growth factor beta family and is highly expressed in several cancers and associated with poor survival [40, 41]. INHBA expression is suggested to correlate with macrophage infiltration in cervical cancer [42] and breast cancer [43]. CSFR1 (Colony stimulating receptor 1) showed a strong correlation to VS size. CSF1R is a type III receptor tyrosine kinase (RTK) that is involved in the proliferation, differentiation, survival, motility, and function of myeloid cells and in promoting disease progression in various conditions ranging from inflammation to cancer [44, 45]. The role of INHBA and CSFR1 in the pathophysiology of VS will be another area of future research.

There is limited information available in literature on the cellular profile of VS using single-cell analysis. Previous work has focused on single-cell analysis of Schwann cells and the interactions between Schwann cells and fibroblasts [46, 47]. Our scRNAseq data further confirms the presence of several cellular subsets in VS including Schwann cells, fibroblasts and immune cells, such as macrophages and T cells. In this work we have focused on macrophages on scRNAseq and show a distinct cluster of macrophages on scRNAseq among the cell types identified and confirm the expression of CD14, CSFR1, INHBA and IL-1β in the macrophages at the single-cell level in addition to the tissue on RT-qPCR. We also found that the macrophages could be grouped into easily identifiable three subclusters based on expression of CD68, CD163 and IL-1β. Of note, IL-1β is a key inflammatory cytokine and has recognised roles in tumour pathogenesis [48]. IL-1β expressing macrophages were found in abundance in VS tissue on immunohistochemistry indicating an important role of IL-1β expressing macrophages in the VS microenvironment.

CD68+CD163+IL-1β+ macrophages showed high expression of Amphiregulin (AREG), PLAUR (Urokinase plasminogen activator surface receptor) and AFF3 (AF4/FMR2 family member 3). AREG is a ligand of the epidermal growth factor receptor (EGFR) [49] and is known to be significantly expressed in M1 macrophages [50]. AREG is an autocrine growth factor as well as a mitogen for a broad range of target cells including astrocytes, Schwann cells and fibroblasts [51]. Its expression primarily in IL-1β producing CD68+CD163+ macrophages makes it an attractive potential target for modulating macrophage behaviour. This group of macrophages also expressed higher levels of PLAUR (Plasminogen Activator Urokinase Receptor) which links with increased macrophage infiltration and poor prognosis in gliomas [52]. Its role in VS behaviour is not yet known and will be an important facet for future research. IL-1β-producing CD68+CD163+ macrophages also expressed AFF3 (AF4/FMR2 family member 3, or LAF4), which encodes a tissue‐restricted nuclear transcriptional activator that is possibly involved in lymphoid cell development [53] and has been identified as an important player in the onset and development of cancers including glioblastoma [54]. The CD68+CD163+IL-1β+ subset also expresses VEGF. This is of interest as in a model of peripheral arterial disease, autocrine IL-1β signalling promoted transcription of pro-angiogenic VEGF via activation of STAT3 and NF-kB [55].

In contrast to the CD68+CD163+IL-1β+ macrophages, CD68+CD163+IL-1β− macrophage subcluster expressed PLCG2 (phospholipase C gamma 2) which is predominantly expressed in hematopoietic cells in the periphery [56] and microglia in the central nervous system (CNS) [57, 58] and NCKAP5 (NCK associated protein 5). NCKAP5 is predicted to be involved in microtubule bundle formation and microtubule depolymerisation but its role in macrophage function specifically is not yet known. Interestingly, this subcluster of macrophages was also found to express S100B which is glial-specific and is expressed primarily by astrocytes, and in the developing CNS it acts as a neurotrophic factor and neuronal survival protein [59].

S100 is also known to be produced by other cell types such as monocytes, macrophages, microglia and T cells [60]. Its production by macrophages in VS microenvironment could promote tumour cell survival. Of interest, in a mouse model of uveoretinitis S100B was seen to increase the expression of IL-1β by macrophages [61]; thus the S100B expression by the subcluster of macrophages and Schwann cells could result in a paracrine effect and IL-1β production by macrophages in the VS microenvironment.

Subcluster 2 of macrophages expressed CD68 but not CD163 suggesting a M1 phenotype. They also expressed IL-1β and showed high expression of AUTS2 (Autism susceptibility candidate 2), SPP1 (secreted phosphoprotein 1 or osteopontin) and Serpine 1 (Plasminogen activator inhibitor-1). AUTS2 is a crucial gene associated with neuropsychological disorders such as epilepsy [62] though its role in macrophage function is yet to be defined. Osteopontin is a secreted glycoprotein that can generate macrophage accumulation [63] and enhance tumour invasion [64]. Serpine 1 promotes the recruitment and polarisation of macrophages in cancer [65]*.* In gliomas, Serpine 1 is closely associated with infiltrations of immune cells in the tumour microenvironment and acts synergistically with PD1, PD-L1, PD-L2 [66]. The presence of three identifiable subsets of macrophages in VS microenvironment is interesting as it demonstrates the heterogeneity of the tumour-infiltrating macrophages and raises the possibility of complex roles of macrophage subsets in VS behaviour. We also show that the VS macrophage subsets are functionally different on GO pathway analysis with Cluster 0 involving pathways in leucocyte migration and response to LPS while Cluster 2 involves pathways in heat response and cytokine activity. Interestingly, Cluster 1 involved predominantly potassium and voltage gated channels pathway. Potassium channels and other voltage gated channels have recently been shown to have important roles in macrophage function such as iNOS production, phagocytosis and intracellular signalling [67]. Transcriptional activity (DoroThea) promoting tumourigenesis and regulation of inflammation was also different between the three clusters of macrophages suggesting that they could influence tumour growth and tumoural inflammation. The VS macrophages were compared with tumour associated macrophages in GM described by Cui et al. [30]. Very few macrophages were noted to be of microglial origin in VS (as assessed by TMEM119 expression) compared to GM. This may be because VS is derived from cranial nerve tissue and is anatomically outside the brain, though intracranial in location; while GM is derived from brain tissue and is a malignant tumour based on pathology while VS is not. Expression of MHCII (marker of activation in macrophages) was present on all clusters of the VS macrophages with highest expression on Cluster 1 (representing the most activated VS macrophage subcluster) and expression of ANKRD28 (marker of repressed macrophages in GM) only in Cluster 0 (representing a potential repressed state). This is in contrast to GM macrophages, where repressed macrophages exhibited absence of both MHCII and ANKRD28 [30]. This shows qualitative differences in the activation/repression status of VS macrophages compared to GM macrophages which may potentially link to different tumour behaviours.

A potential limitation of our study is that while sporadic VS can present between the ages of 30 and 70 years, the scRNAseq reported in this study has been performed in three patients in the younger age group compared to the average age at presentation. IL-1β-positive macrophages were, however, found in tumours from older patients as well. It will be interesting to explore in future studies if age of the patient has an impact on the macrophage profile in VS. There is currently no evidence in literature that sporadic VS tumours have a different clinical course in younger versus older patients though it is possible that surgical intervention is likely to be favoured in the younger patient group compared to the older cohort due to better tolerance to anaesthesia and prolonged surgery in the younger patients.

In summary, our results show that VS tissue express several molecules involved in macrophage recruitment and function that correlate to the tumour volume. We also show using scRNAseq that three separate macrophage subtypes in the VS environment can be identified with distinct profiles and differential expression of CD68, CD163 and IL-1β. This opens the possibility of selectively targeting macrophage populations that contribute to VS tumour growth and avoiding non-specific effects on potentially ‘beneficial’ macrophage populations. Further understanding of the roles of these individual molecules and macrophage subtypes in VS pathogenesis could reveal novel targets to block tumour growth and bring about a step change in the treatment of VS.

### Supplementary information

Supplemental material

## Data Availability

The transcriptomics data included in this manuscript has been made available on the GEO platform and full code will be made available on request. The details are in the ‘Materials and methods’ section.
