# Supplementary material for: Single-cell RNA sequencing analysis of vestibular schwannoma reveals functionally distinct macrophage subsets
Source: Br J Cancer. 2024 Mar 13;130(10):1659–69. doi: 10.1038/s41416-024-02646-2 (PMC11091088; doi:10.1038/s41416-024-02646-2)
Supplement: Supplementary file 1 — Supplemental material [file 41416_2024_2646_MOESM1_ESM.docx]

**Supplementary data**

**Supplementary Figure 1**

**
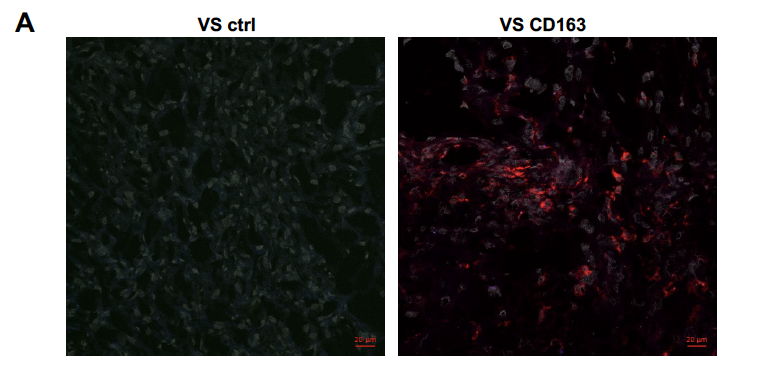
**

**
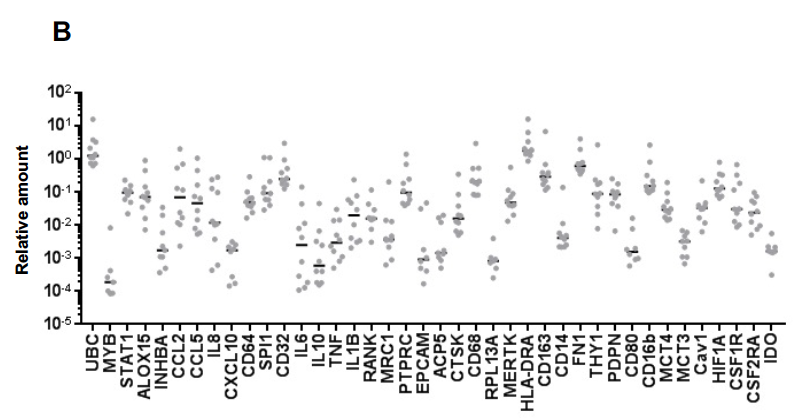
**

**Supplementary Figure 2**

**
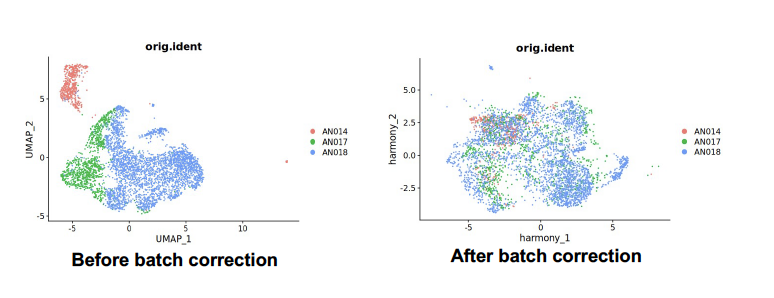
**

**Supplementary Figure 3**

**
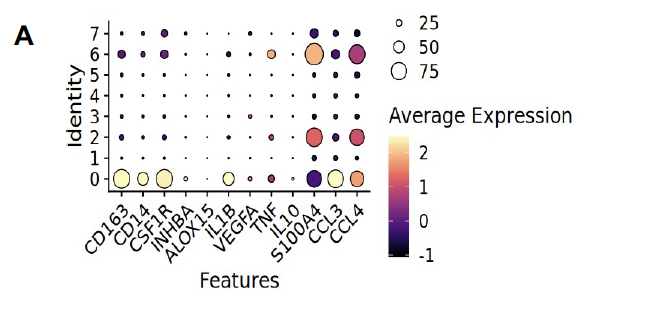
**

**
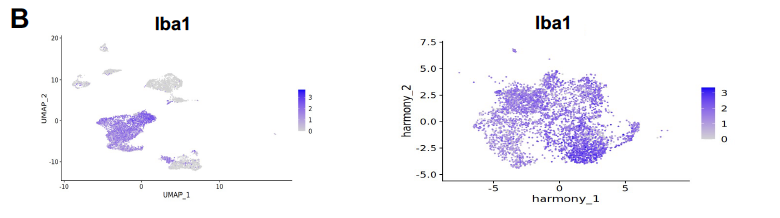
**

**
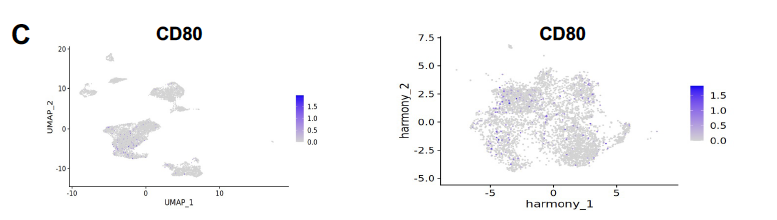
**

**
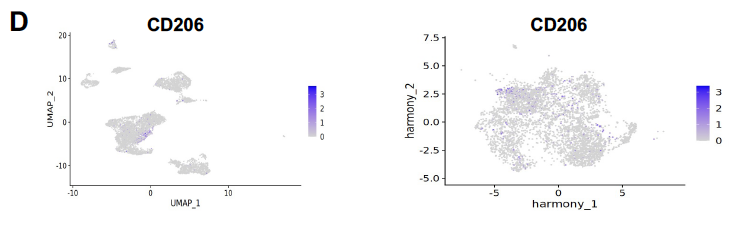
**

**Supplementary Figure 4**

**
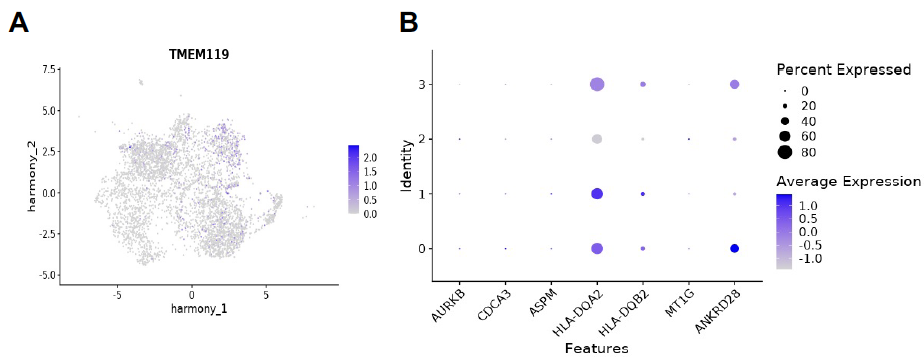
**

**Supplementary Legends**

**Supplementary Figure 1A: Expression of CD163 in vestibular schwannoma (VS) tissue on immunofluorescence**

Fresh frozen VS sections were imaged using immunofluorescence to assess CD163 expression. Isotype control antibodies staining is on the upper left panel. Samples on the right upper panel and lower panels were stained with antibodies against human CD163 (red). Nuclei are grey. Representative samples from three different patients are shown.

**Supplementary Figure 1B: RT-qPCR analysis of fibroblast associated molecules in vestibular schwannoma (VS)** Expression of a panel of macrophage genes on RT-qPCR in fresh frozen tissue from 10 human VS samples. Each data point is one patient sample and shows the relative amounts for each gene using GAPDH as the housekeeping gene. Correlation of the expression levels to tumour volume is presented in Table 2, 3 and 4.

**Supplementary Figure 2: Macrophage subclusters on scRNA analysis.** Single cell RNA analysis of the macrophage subsets in three vestibular schwannoma tissue samples from different patients. Macrophage cluster before (left panel) and after batch correction (right panel).

**Supplementary Figure 3: Expression of macrophage markers in VS macrophage subclusters on scRNA analysis.** Single cell RNA analysis of the macrophage subsets in three vestibular schwannoma tissue samples from different patients. **A)** macrophage cluster before and after batch correction. **B)** Expression of Iba1 in whole cell population (left panel) and macrophage cluster (right panel). **C)** Expression of CD80 in whole cell population (left panel) and macrophage cluster (right panel). **D)** Expression of CD206 in whole cell population (left panel) and macrophage cluster (right panel).

**Supplementary Figure 4: Expression of microglial and activation markers in VS macrophage subclusters on scRNA analysis.** Single cell RNA analysis of the macrophage subsets in three vestibular schwannoma tissue samples from different patients. **A)** Expression of TMEM119 in the macrophage subsets. **B)** Expression of aurora kinase B (AURKB), cell division cycle associated 3 (CDCA3), assembly factor for spindle microtubules (ASPM), HLA-DQA2, HLA-DQB2, Metallothionein (MT1G) and ankyrin repeat domain 28 (ANKRD28).

**Supplementary methods**

**Human tissue processing and histology**

Tumour samples were frozen in Tissue-Tek OCT medium. For immunofluorescence, acetone-fixed frozen sections were incubated with mouse anti-human-CD163 (Clone GHI/61, BD Pharmingen). Control antibodies used for staining were mouse IgG1 (DAKO). These were detected with goat anti-mouse IgG 546 (Molecular Probes). Images were acquired with a Leica DM6000B and analysed using Zen software.

Paraffin embedded tumour samples were used for immunohistochemistry. Antigen retrieval was performed at pH9 for 20 minutes. Sections were stained using polyclonal rabbit anti-human IL-1β (Proteintech 16806-1-AP, conc: 1:500) and mouse anti-human CD163 (Leica Clone -10D6, conc 1:400) and mouse anti-human CD68 (Leica Clone - 514H12, conc 1:100) for 30 minutes at room temperature. For the DABs and Red staining, Leica Bond Polymer Refine Detection systems were used. Nuclei were counterstained with Hematoxylin QS (Vector Laboratories). Images were acquired using the Zeiss Axio Scan and analysed with QuPath software (v0.4.3).

**Data deposition**

Data is deposited under: GSE250061. Code is available: chrismahony/Baruah-et-al.-BJC-2023: Baruah et al., BJC, 2023 (github.com)

**Supplementary Table 1**

**
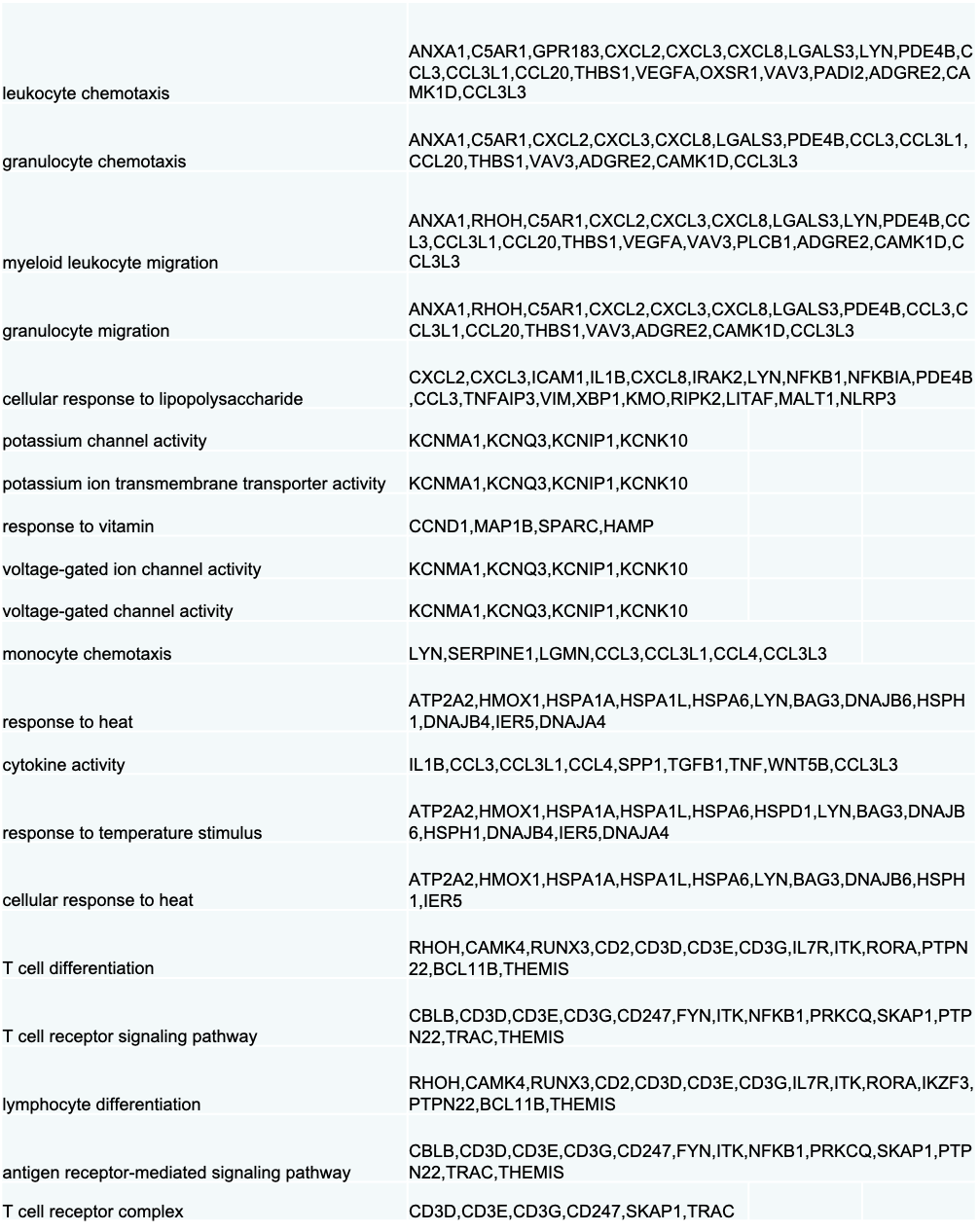

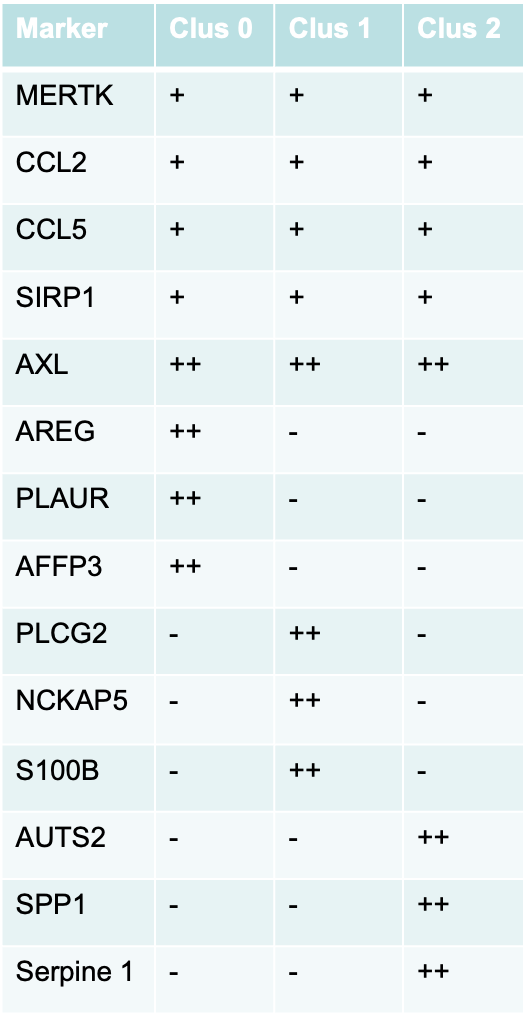

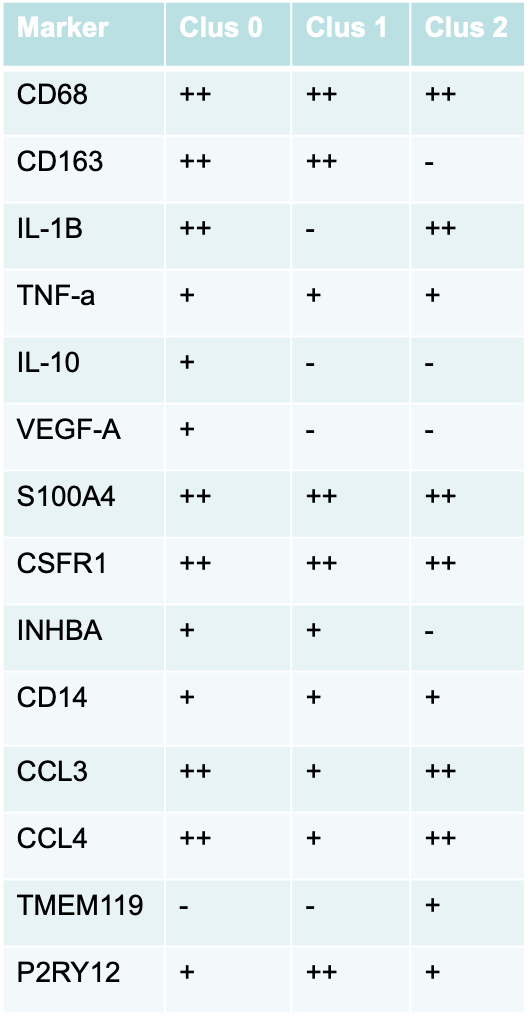
**

**Supplementary Table 2:**

**Expression of markers in macrophage subclusters**
